# Contralateral reexpansion pulmonary edema with ipsilateral collapsed lung after pleural effusion drainage: a case report

**DOI:** 10.1186/s13019-015-0272-3

**Published:** 2015-05-08

**Authors:** Jae Jun Kim, Yong Hwan Kim, Si Young Choi, Seong Cheol Jeong, Seok Whan Moon

**Affiliations:** 1 Department of Thoracic and Cardiovascular Surgery, College of Medicine, St. Mary’s Hospital, Uijeongbu, South Korea; 2 Department of Thoracic and Cardiovascular Surgery, College of Medicine, St. Mary’s Hospital, Seoul, South Korea

**Keywords:** Reexpansion pulmonary edema, Malignant pleural effusion, Thoracostomy

## Abstract

Reexpansion pulmonary edema is a rare but potentially life-threatening condition that occurs when a collapsed lung reexpands, usually in the same side of collapsed lung. We present a rare case in which a 57-year-old Korean man had a large amount of malignant pleural effusion. After undergoing tube thoracostomy drainage for the pleural effusion, a contralateral reexpansion pulmonary edema developed while the ipsilateral lung was half collapsed. The patient was dyspneic with an oxygen saturation that dropped to 66 %. After conservative treatment with oxygen therapy, steroid administration, and negative suction application (suction pressure of -20 cm H_2_O) in the right pleural cavity for five days, the right lung could be fully expanded without development of reexpansion pulmonary edema, and the reexpansion pulmonary edema in the left lung resolved. Although it is a very rare condition, it is important to know that contralateral occurrence of reexpansion pulmonary edema can occur, especially when the ipsilateral lung is collapsed. Being aware of this potential condition can allow for early and proper management.

## Background

Reexpansion pulmonary edema is a rare but potentially life-threatening condition that usually occurs through rapid reexpansion of a chronically collapsed lung [1–3]. It can occur in every type of chronically collapsed lung, including pneumothorax, pleural effusion, or a huge mediastinal mass [1]. The present case describes a 57-year-old Korean man who developed contralateral reexpansion pulmonary edema with the ipsilateral collapsed lung after tube thoracostomy drainage of a malignant pleural effusion.

### Case presentation

**Fig. 1:**
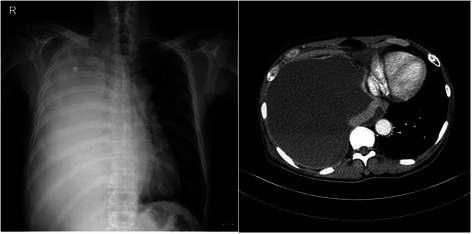
Initial chest x-ray and CT show that massive pleural effusion in the right hemithorax combined with diffuse pleural thickening and several small nodules in the left lung, suggesting malignant pleural effusion

**Fig. 2:**
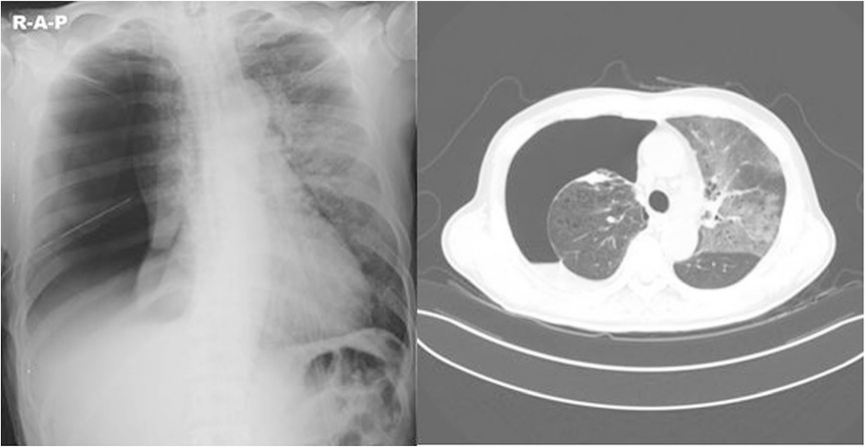
A chest radiograph and chest CT show the right half- collapsed lung, and diffuse and severe infiltrative patterns suggesting reexpansion pulmonary edema in the left whole lung field

A 57-year-old Korean man with no underlying disease visited our hospital emergency department for progressive dyspnea over the course of several days. The initial history revealed he was conscious, alert, and oriented. Vital signs showed that the patient was tachypneic (39/min), tachycardic (115/min) and normotensive (130/90 mm Hg). However, he was not hypoxic (O_2_ saturation 98 % on room air). Laboratory findings were non- specific. Initial chest x-ray and CT showed massive pleural effusion in the right hemithorax, diffuse pleural thickening, and several small nodules in the left lung, suggesting malignant pleural effusion (Fig. 1). At first, an 8.3- French pig tail catheter drainage for the pleural effusion was performed. The pleural effusion study showed sanguineous and lympho-dominant exudate. Carcinoembryonic antigen level was 367.6 μg/L and adenosine deaminase was measured at 41 IU/L. However, the cytology findings of the effusion were negative for malignancy. Because the pleural effusion was not effectively drained, a 24-French tube thoracostomy without any negative pressure suction was performed in the right pleural cavity. The initial amount of drainage was 1500 ml. The patient tolerated the procedure well and his symptoms improved. However, the patient’s dyspnea worsened, and his oxygen saturation dropped to 66 % on room air the next morning. The patient required a 100 % FiO_2_ via mask with a reservoir bag to maintain and stabilize his oxygen saturation. A chest radiograph and chest CT taken at that time showed a right half- collapsed lung, and diffuse and severe infiltrative patterns, suggesting reexpansion pulmonary edema in the left whole lung field (Fig. 2).

After conservative treatment with oxygen therapy, steroid administration, and negative suction application (-20 cm H_2_O) in the right pleural cavity for five days, the right lung could be fully expanded without the development of ipsilateral reexpansion pulmonary edema and the reexpansion pulmonary edema in the left lung resolved. Fortunately, the negative suction application (-20 cm H_2_O) in the right pleural cavity did not lead to development of reexpansion pulmonary edema or exacerbate the clinical course. The patient was weaned from oxygen supplementation.

For accurate diagnosis to determine proper management, we performed a VATS biopsy of the right pleura on the 10^th^ hospital day. The operative findings showed diffuse emphysematous lung, diffuse pleural nodularity, and sanguineous pleural effusion. The final pathology revealed a primary adenocarcinoma lung cancer with pleural metastasis. The patient’s postoperative course was uneventful. He is now undergoing palliative chemotherapy.

## Discussion

Reexpansion pulmonary edema is usually associated with reexpansion of a chronically collapsed lung due to a large amount of air, pleural fluid, or huge masses [1–3]. The reexpansion pulmonary edema mostly happens within a few hours after reexpansion of the collapsed lung [1]. However, the onset of reexpansion pulmonary edema can occur anytime within 24 h of reexpansion [1]. Reexpansion pulmonary edema is a rare but potentially life- threatening condition (mortality of up to 20 %) [2]. The clinical manifestations of reexpansion pulmonary edema can range from radiographic findings without any clinical symptoms to fetal hypoxia or even hemodynamic instability [2]. Because most cases are detected incidentally on radiography, the true incidence is still unclear (0.9 -20 %) [2]. The chest CT findings of reexpansion pulmonary edema are peripheral patchy lesions of ground glass opacity with a vascular distribution, which were usually associated with consolidation as well as interstitial thickening [2–4].

Although exact etiologies and mechanisms are still not fully understood, some pathogenic factors for reexpansion pulmonary edema have been suggested: (1) rapid reexpansion, (2) drainage with the use of negative intrapleural pressure, (3) decreased surfactant activity, (4) increased pulmonary vascular permeability due to injuries to the pulmonary micro- vessels, (5) airway obstruction, (6) pulmonary artery pressure change, and (7) chronicity of lung collapse [1, 2, 4, 5]. Rapid reexpansion, drainage with the use of negative intrapleural pressure, and chronicity of lung collapse are considered the major risk factors for reexpansion pulmonary edema [4].

The mainstay of treatment remains generally conservative and supportive, including sufficient oxygen supplement [1–3]. Some more intensive treatments, such as mechanical ventilation, steroid and diuretics administration, and circulation resuscitation, are required in severe cases of reexpansion pulmonary edema, such as treatment of adult respiratory distress syndrome [1–3]. Concomitant contralateral reexpansion pulmonary edema is associated with more severe symptoms and higher mortality [6].

Mostly, reexpansion pulmonary edema is limited to the ipsilateral collapsed lung after relief of collapse. Occurrence of reexpansion pulmonary edema in the contralateral non-collapsed lung is also possible, though very rare [6]. Possible hypotheses for contralateral reexpansion pulmonary edema have been suggested: (1) oblivious aspiration, (2) compressive forces due to severe mediastinal shift, (3) systemic inflammatory response followed by reexpansion, especially in a previously injured lung or in those with significant pulmonary disease, and (4) significant increased cardiac output after rapid reexpansion of lung [6].

Like the present case, contralateral reexpansion pulmonary edema without ipsilateral occurrence is very rare. However, it could be diagnosed by no evidence of aspiration, no fluid overloading, no renal and cardiac failure, radiologic findings compatible with reexpansion pulmonary edema, excellent response to steroid therapy, and no signs of infection.

Unlike previous reports in the literature, the present case is unique in that it presents contralateral reexpansion pulmonary edema with ipsilateral collapsed lung. Because the ipsilateral lung was not fully expanded but significantly collapsed, and nearly all pleural effusion was rapidly drained, compressive forces due to severe mediastinal shift in the contralateral lung were more relieved than in condition of full expansion of ipsilateral lung. Therefore, the changes associated with the pathogenesis of reexpansion pulmonary edema are more prominent in the contralateral lung. In addition, there was severe pulmonary emphysema in both lung fields.

The hypotheses of rapid reexpansion and compressive forces due to severe mediastinal shift by ipsilateral pleural effusion drainage of the collapsed lung and high incidence in the pulmonary disease could be proved in this present case. In addition, negative intrapleural suction of the collapsed right lung is used for the reexpansion for the collapsed right lung and for patient stabilization. Fortunately, the right lung could be reexpanded without development of ipsilateral reexpansion pulmonary edema or aggravation of the contralateral reexpansion pulmonary edema. However, the use of negative pressure suction for reexpansion of the ipsilateral collapsed lung with the presence of contralateral reexpansion edema is controversial because it is not known if negative pressure suction could aggravate the ipsilateral, contralateral, or bilateral reexpansion edema.

## Conclusion

In spite of the rare incidence of reexpansion pulmonary edema, it is important to know that contralateral re-expansion pulmonary edema can occur, especially when the ipsilateral lung is collapsed. Being aware of this potential condition can allow for early and proper management.

### Informed consent

Written informed consent was obtained from the patient for publication of this Case report and any accompanying images. A copy of the written consent is available for review by the Editor-in-Chief of this journal. This case study was approved by Institutional Review Board for Uijeongbu St. Mary’s Hospital (UC14ZISE0156).
